# Six weeks of polarized functional interval training with large training load reductions does not affect performance gains compared to traditional workouts

**DOI:** 10.3389/fphys.2024.1446837

**Published:** 2024-11-15

**Authors:** Steffen Held, Eduard Isenmann, Ludwig Rappelt, Tim Wiedenmann, Dominic Kutschki, Jannik Harbrecht, Katrin Kirchner, Stephan Geisler, Lars Donath

**Affiliations:** ^1^ Department of Sport and Management, IST University of Applied Sciences, Duesseldorf, Germany; ^2^ Department of Intervention Research in Exercise Training, German Sport University Cologne, Cologne, Germany; ^3^ Department of Fitness and Health, IST University of Applied Sciences, Duesseldorf, Germany; ^4^ Institute for Cardiovascular Research and Sports Medicine, German Sport University Cologne, Cologne, Germany; ^5^ Department of Movement and Training Science, University of Wuppertal, Wuppertal, Germany

**Keywords:** circle training, training intensity distribution, VO2max, endurance, strength

## Abstract

**Purpose:**

High-intensity functional interval training (HIFT) is predominantly composed of high exercise training intensities (HiT) and loads. Both have been linked to a higher risk of overtraining and injuries in inexperienced populations. A polarized training approach is characterized by high amounts of low-intensity training (LiT) and only approximately 5%–20% HiT. Compared to HIT-based training, this approach can result in temporary training load and intensity reductions without diminishing training gains. Thus, we aimed to examine the effects of traditional (TRAD) HIFT vs. polarized (POL) HIFT on relevant performance parameters.

**Methods:**

Thirty athletes (15 females, age: 26.6 ± 5.0 years, height: 1.76 ± 0.13 m, body mass: 79.6 ± 12.4 kg, prior experience: 2.3 ± 2.0 years, training volume: 6.1 ± 2.4 h/wk) were randomly assigned to 6 weeks of either POL (78% LiT, 22% threshold intensity training (ThT) to HiT) or TRAD (26% LiT, 74% ThT to HiT). HIFT performance testing focused on maximal strength (squat: SQ1RM, deadlift: DL1RM, overhead press: OHP1RM, high pull: HP1RM), endurance (peak oxygen uptake: V̇O_2_peak, lactate threshold: LT, peak power output (PPO), and benchmark HIFT workout (Jackie: 1000 m rowing, 50 thrusters, and 30 pull-ups for time).

**Results:**

POL (785 ± 71 au) completed significantly (*p* ≤ 0.001; SMD = 4.55) lower training load (eTRIMP) than TRAD (1,273 ± 126 au). rANCOVA revealed no statistical relevant group×time interaction effects (0.094 ≤ *p* ≤ 0.986; 0.00 ≤ η_p_^2^ ≤ 0.09) for SQ1RM, DL1RM, OHP1RM, high pull, V̇O2peak, LT, PPO, and Jackie performance. Both groups revealed trivial to moderate but significant (rANCOVA time effects: *p* ≤ 0.02; 0.01 ≤ η_p_^2^ ≤ 0.11; 0.00 ≤ SMD ≤ 0.65) performance gains regarding DL1RM, OHP1RM, HP1RM, and Jackie.

**Conclusion:**

Despite a notably lower total training load, conditioning gains were not affected by a polarized functional interval training regimen.

## 1 Introduction

High-intensity functional interval training (HIFT) is the basis of CrossFit®, which has developed into a widely popular sport (Claudino et al., 2018; Dominski et al., 2022; Fisker et al., 2017) aiming at optimizing a wide array of physical performance measures, such as strength, endurance, stamina, flexibility, power, speed, coordination, agility, balance, and accuracy domains (Glassman, 2002). With a focus on varying functional movements, HIFT training incorporates key elements of gymnastics (e.g., handstand and ring exercises), weightlifting exercises (e.g., barbell squats and presses), and traditional cardiovascular activities (e.g., running or rowing) as exercises (Fisker et al., 2017). These HIFT exercises are typically performed quickly, repeatedly, and with comparatively high training intensity, while the inter-set recovery time is reduced (Sprey et al., 2016). Consequently, apart from strength improvements (Ambroży et al., 2022), an increased maximal rate of oxygen consumption (V̇O_2_max) has been observed through a HIFT-based training approach (Eather et al., 2016).

Previous studies have identified training intensity and its distribution as crucial parameters that can be manipulated to alter performance markers (Meeusen et al., 2006). High-intensity training has been shown to improve endurance performance relative to low-intensity training for various key endurance measures such as V̇O_2_max, time-trial performance, exercise economy, and time-to-exhaustion (Helgerud et al., 2007; Seiler et al., 2013).

However, a high volume of high-intensity training can adversely affect recovery, leading to reduced performance, disturbed sleep, increased perceived fatigue, and a higher incidence of respiratory tract infections (Hausswirth et al., 2019; Le Meur et al., 2013). The recovery is not necessarily compromised by high loads, unless the recovery period is insufficient to balance the increased training stress. The relationship between stress, strain, recovery, and adaptation is crucial—recovery that is inadequate relative to the imposed training stress, particularly with heavy or high-intensity loads, can impair adaptation and increase the risk of negative outcomes such as overtraining, injury, or illness (Meeusen et al., 2006). Thus, appropriate periodization and recovery strategies are essential in mitigating these risks, while optimizing performance gains.

Therefore, the training schedule should ensure the frequency of high-load training provides the required recovery time, and the intensity distribution of each session also varies in a manner to support effective recovery to avoid severe fatigue, stagnation, and possibly overtraining (Rosenblat et al., 2019).

The intensity distribution of a training session is the volume performed at various specified training intensities. Several studies have examined the intensity distributions employed by endurance athletes (Sperlich et al., 2023). These studies on sports such as cycling, rowing, skiing, biathlon, running, swimming, speed skating, and triathlon have reported approximately 75%–85% of the total training volume is performed in the low-intensity zone, up to 20% in the moderate-intensity zone, and up to 10% in the high-intensity zone (Esteve-Lanao et al., 2005; Muñoz et al., 2014; Neal et al., 2011; Seiler and Kjerland, 2006). This training intensity distribution (TID) has been previously described as a pyramidal (PYR) or polarized (POL) training model (Seiler and Kjerland, 2006; Sperlich et al., 2023). The POL model is characterized by approximately 80%–95% LiT (below the first lactate threshold) and approximately 5%–20% HiT (above the second lactate threshold) while avoiding the moderate threshold-based intensity zone (ThT, between the first and second lactate thresholds) as much as possible (Röhrken et al., 2020; Seiler and Kjerland, 2006; Sperlich et al., 2023). Regarding the PYR model, slightly more moderate-intensity training is included than high-intensity training, resulting in approximately 60%–90% LiT, 5%–30% ThT, and 2%–10% HiT (Röhrken et al., 2020; Seiler and Kjerland, 2006; Sperlich et al., 2023). Both models are characterized by (very) high volumes of low-intensity training (Esteve-Lanao et al., 2005; Muñoz et al., 2014; Neal et al., 2011; Seiler and Kjerland, 2006). In contrast, the threshold training intensity distribution model differs from the PYR and POL model, in that a significant percentage of training (35%–55%) is completed in the moderate-intensity zone, with a smaller percentage of training (45%–55%) completed in the low-intensity zone (Seiler and Kjerland, 2006). Furthermore, the HiT-based approach, where high-intensity training constitutes more than 30%–50% of the total training time, is rarely used in elite endurance sports (Sperlich et al., 2023).

Based on the winning times of individual events (i.e., duration from start to finish) at the CrossFit® Games (2017–2021), the average load time is 9.0 min (95% confidence interval (95%CI) 1.4–11.6 min) for men and 8.8 min (95%CI: 1.4–11.5 min) for women. Similarly, the normative scores of the CrossFit® open workout between 2011 and 2022 revealed similar average load times (Mangine et al., 2023). Given the duration and intensity required to maintain a high level of performance throughout these events, we can infer that CrossFit® competition primarily engages both aerobic and anaerobic energy systems, placing it within the realm of endurance-based activities. In sports with similar energy system demands and load times (in competition), athletes often utilize TID heavily focused on LiT, with at least 80% of LiT (Seiler and Kjerland, 2006; Seiler, 2010; Sperlich et al., 2023; Stöggl and Sperlich, 2014). Consequently, reviews (Hydren and Cohen, 2015; Seiler and Kjerland, 2006; S; Seiler, 2010; Stöggl and Sperlich, 2014) have suggested that a polarized (POL) TID may elicit superior training adaptations than high-intensity-focused approaches, particularly in endurance sports.

Against this background, we examined the effect of polarized vs. traditional HIFT training on relevant CrossFit® performance surrogate parameters. Based on previous endurance sports-related reviews and meta-analyses (Hydren and Cohen, 2015; Rosenblat et al., 2019; Seiler and Kjerland, 2006; Seiler, 2010; Stöggl and Sperlich, 2014), we hypothesized that these findings could be transferred to a HIFT training setting and may impact the programming in HIFT. They may necessitate a re-evaluation of current training paradigms, potentially leading to a shift in how HIFT training is structured and implemented. This shift toward a more polarized training intensity distribution approach could influence not only the effectiveness and efficiency of training but also aspects related to athlete health, injury prevention, and long-term athletic development within the HIFT community.

## 2 Methods

### 2.1 Participants

Based on a previous meta-analysis on polarized training (Rosenblat et al., 2019), an *a priori* power analysis (α = 0.05, study power (1−β-error) = 0.80, effect size partial eta squared (η_p_^2^) = 0.06 (f = 0.26), correlations among repetitive measures = 0.6; g*Power, Version 3.1.9.6) (Faul et al., 2007) revealed a required sample size of n = 28. Assuming a moderate dropout rate, 30 trained HIFT athletes (Table 1) were enrolled in the present randomized controlled interventional trial. The participant recruitment period ranged from 1 December 2022 to 1 March 2023. All participants were at least 18 years of age, had a previously weekly training volume of at least three HIFT training sessions per week, showed no health impairments, and were familiarized with the test and training procedures prior to the start of the study. We recorded the phase of the menstrual cycle for female participants and ensured that testing did not occur during menstruation. The study protocol complied with the Declaration of Helsinki and was approved by the local ethical committee (144/2022). International ethical standards were met (Harriss and Atkinson, 2015), and all participants signed an informed written consent after receiving all relevant study information. In addition, both groups did not differ (*p* ≥ 0.118; SMD ≤ 0.61) regarding height, age, body mass, experience, and prior training volume (Table 1).

**TABLE 1:** Anthropometric data of the polarized (POL) and the traditional (TRAD) HIFT training groups.

Parameter	POL	TRAD	t-test [p (SMD)]
Sample size	n = 16 (8 females)	n = 14 (7 females)	---
Age (yrs)	Total: 25.3 ± 4.6 Females: 23.9 ± 3.6 Males: 27.0 ± 5.4	Total: 28.2 ± 4.6 Females: 29.4 ± 5.1 Males: 27.4 ± 4.4	0.118 (0.61)
Height (cm)	Total: 175.9 ± 10.6 Females: 169.9 ± 9.2 Males: 182.9 ± 7.6	Total: 178.8 ± 10.1 Females: 171.6 ± 6.1 Males: 183.4 ± 9.7	0.465 (0.28)
Body mass (kg)	Total: 81.1 ± 15.1 Females: 78.3 ± 16.8 Males: 80.6 ± 11.0	Total: 78.1 ± 10.6 Females: 69.7 ± 12.5 Males: 82.2 ± 7.4	0.396 (0.17)
Experience (yrs)	Total: 2.0 ± 1.8 Females: 1.7 ± 1.0 Males: 2.6 ± 2.5	Total: 2.6 ± 1.7 Females: 1.7 ± 0.7 Males: 3.1 ± 2.0	0.478 (0.27)
Prior training (h/wk)	Total: 6.6 ± 1.8 Females: 6.5 ± 3,4 Males: 6.6 ± 2.5	Total: 6.4 ± 1.8 Females: 5.7 ± 1.8 Males: 6.4 ± 1.9	0.652 (0.17)

Data are given as mean ± standard derivation. In addition, *p*-values of independent t-tests and standardized mean differences (SMD) are given as pairwise effect sizes.

### 2.2 Study design

This study was designed as a randomized controlled trial with a parallel group design. All included athletes were randomly assigned either to a time-matched polarized (POL) or usual (TRAD) HIFT training group via *minimization* (Scott et al., 2002). Thereby, gender, age, height, body mass, and peak power output were used as strata. Participants were instructed to avoid any strenuous exercise 2 days before each testing session. To control for potential circadian interference with performance, all measurements were conducted at similar times of the day for each participant.

### 2.3 Training procedure

During the 6-week intervention period, both groups trained four times weekly using the same exercises. The TRAD group trained as usual following a HIFT training regimen. The POL group employed a polarized training approach. Thereby, the participants aimed to stay below a target heart rate corresponding to the first lactate threshold (Dickhuth et al., 1991) for three of four weekly training sessions. During the fourth training session each week, the participants aimed at meeting the high-intensity zone (i.e., reaching a heart rate above the second lactate threshold (Dickhuth et al., 1991)). The heart rates of both training groups were continuously monitored during the endurance/HIFT training via a chest strap (H9, Polar Electro Oy, Kempele, Finland). Training sessions of both groups were supervised by certificated coaches. The HRs of both groups were monitored and displayed in real-time to the participants via the Polar Teams app (Polar Electro, Kempele, Finland). In addition, the corresponding supervisor ensured that the corresponding individual HR limits were adhered to via verbal feedback. Thereby, live HR feedback was used to ensure that athletes stayed within the required HR zones. Specifically, the movement tempo, workload, or power was adjusted if the HR was too low or too high. Certified coaches designed the training workouts for both groups during pilot work prior to the commencement of the experiment, and they supervised every training session. Detailed descriptions of both training regimes are given in supplemental files. To ensure that the prescribed training regimes were consistently followed, dedicated HIFT classes were established for both training groups in two separate HIFT gyms. These classes were specifically designed to standardize the training sessions, ensuring that all participants completed the same workouts each week. By organizing exclusive classes, we eliminated variability in workout routines and maintained strict adherence to the intervention protocols. This setup mitigated the potential confounding effects of differing day-to-day workouts typically seen in regular HIFT gym/box schedules, thereby preserving the integrity of our results. In line with previous interventional exercise studies (Held et al., 2020; Held et al., 2021; Held et al., 2023; Held et al., 2024), the training data of all endurance/HIFT-related parts were monitored using a three-zone heart rate-based approach (Seiler, 2010): Accumulated training time below the first lactate threshold (low-intensity training; LiT); between the first and second lactate threshold (threshold training; ThT); and above the second lactate threshold (high-intensity training; HiT) were recorded separately. In addition, the training dose was monitored daily via an online platform (PolarFlow, H9; Polar Electro, Kempele, Finland). Because a three-zone model (Seiler and Kjerland, 2006; Seiler, 2010) was used, heart rate-based eTRIMP (Eather et al., 2016) was calculated based on the time spent in three HR zones, multiplied by a zone-specific arbitrary weighting factor, and then summed to provide a total TRIMP score: LiT weighting factor = 1; ThT weighting factor = 2; HiT weighting factor = 3. Furthermore, wellbeing status was recorded via the online platform. Thereby, negative events like physical exhaustion, strain, or injury were reported.

### 2.4 Testing procedure

The testing procedure during pre and post testing was conducted on two separate lab visits. During the first lab visit, individual lactate thresholds and peak oxygen uptake (V̇O_2_peak) were assessed. During the second lab visit, the strength- and HIFT-specific performance data (details are given below) were assessed. Prior to each lab visit, a standardized 10-min warmup of easy cycling (with a heart rate corresponding below 2 mmol/L blood lactate concentration) was performed.

To determine individual lactate thresholds and assess V̇O_2_peak, a combined incremental and ramp testing protocol was conducted on a concentric cycle ergometer (Wahoo Kickr V5 Fitness WF133, Wahoo Fitness, Atlanta, United States) until voluntary exhaustion. Cycling was performed with clipless pedals, and participants were instructed to remain seated. This setup revealed a high intraclass correlation coefficient of 1.00 (95% confidence intervals 1.00–1.00) for reliability measurements with a typical error of 3.1 W and 1.6% (Zadow et al., 2018). The test started at a load of 50 W, which was subsequently increased by 30 W every 3 min until reaching a blood lactate concentration of 4 mmol/L, which was immediately followed by the ramp protocol (starting at last step interval power, 30 W increment per minute). Prior to the start of the test, after each 3-min step, and immediately after exercise cessation, blood lactate samples (20 μL) were obtained from the earlobe (Biosen C-Line; EKF Diagnostic Sales, Magdeburg, Germany). Lactate concentrations of the step test were subsequently plotted against the load (in W) and fitted with a third-order polynomial function. Based on this function, heart rate and power at the first lactate threshold (minimal lactate equivalent; LT1) and second lactate threshold (LT2 = LT1 +1.5 mmol/L) (Dickhuth et al., 1991) were estimated. Heart rates (H9; Polar Electro, Kempele, Finland) and respiratory gas exchange data were continuously recorded via a breath-by-breath system comprising a validated metabolic analyzer (Zan Oxi 600, Zan Messgeräte, Germany). Prior to each measurement, this spirometric system was calibrated following the manufacturer’s recommendations. The highest consecutive oxygen uptake values averaged over 30 s were considered as V̇O_2_peak. All athletes were verbally encouraged in a standardized manner until objective exhaustion. Objective exhaustion level was verified using available exhaustion criteria (Midgley et al., 2007). In addition, the reached power during this testing procedure was defined as peak power output (PPO).

To determine the one-repetition maximum (1RM) of the squat, deadlift, overhead press, and high pull, a repetition maximum (XRM) test for each exercise was performed for each exercise using the Lombardi (Lombardi, 1989) formula (CV = 3.4%, ICC = 0.94) (García-Ramos et al., 2019). During this XRM testing (Lombardi, 1989), a training set was performed with 95% of the presumed 1RM until failure (Steele et al., 2017). Participants performed two warm-up sets with approximately 30%–40% and 50%–60% of the presumed 1RM prior to the testing set. During these XRM testing procedures, the corresponding HIFT competition standards that define technical movement execution, such as squat deep below parallel, were applied. All strength tests were supervised by certificated strength coaches. Subsequently, HIFT-specific performance was assessed via the benchmark workout “Jackie” (Mangine et al., 2018). This HIFT-based benchmark workout consisted of completing 1,000 m rowing (Concept2/Type D, Morrisville, United States), 50 thrusters (males: 20 kg; females: 15 kg), and 30 pull-ups as fast as possible.

### 2.5 Statistics

Data are presented as means ± standard deviation. Normal distribution was verified via the Shapiro–Wilk test (*p* ≥ 0.1). Variance homogeneity was visually verified via residual plotting (Kozak and Piepho, 2018). Separate independent t-tests were computed to examine differences in anthropometric (age, height, body mass, experience, and prior training volume) and training data (LiT, ThT, HiT, total training time, and eTRIMP) of POL vs. TRAD. Several separately conducted 2 (group: POL vs. TRAD) × 2 (time: PRE vs. POST) repeated measurement variance analyses with covariate (rANCOVAs) (Vickers and Altman, 2001) were computed for V̇O_2_peak, lactate threshold, peak power output, Jackie, squat 1RM, high pull 1RM, deadlift 1RM, and overhead press 1RM using baseline (pre) test parameters as covariates. rANCOVA effect sizes were given as partial eta-squared (η_p_^2^) with ≥0.01, ≥0.06, and ≥0.14 indicating small, moderate, and large effects, respectively (Cohen, 1988). In the case of significant group × time interaction effects, Bonferroni post-hoc tests were subsequently computed. For pairwise effect size comparison, standardized mean differences (SMD) were additionally calculated (trivial: SMD < 0.2, small: 0.2 ≤ SMD < 0.5, moderate: 0.5 ≤ SMD < 0.8, and large SMD ≥ 0.8) (Cohen, 1988). All statistical analyses were conducted using R (version 4.0.5) and RStudio (version 1.4.1106) software. Wellbeing status was evaluated using a contingency table for the incidence of poor wellbeing, physical overexertion, or injury in POL vs. TRAD groups. Thereby, a Fisher’s exact test was conducted to determine the significance of the differences.

## 3 Results

### 3.1 Training data

**FIGURE 1:**
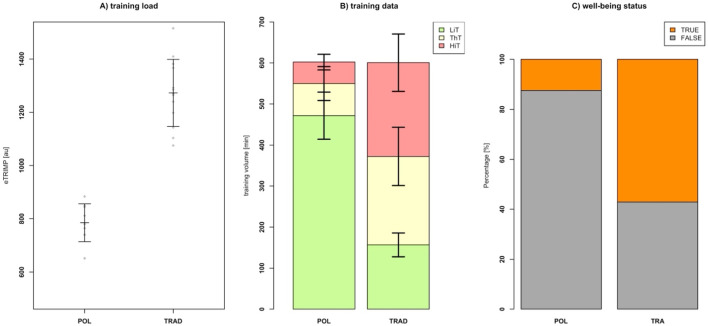
Training load **(A)**, training distribution **(B)**, and wellbeing status **(C)** data of the polarized (POL) and the traditional (TRAD) HIFT training groups. Thereby, training impulse (eTRIMP) values are given as means with standard derivations. Individual data are plotted as grey dots. Regarding the training distribution data, low-intensity training (LiT), threshold training (ThT), and high-intensity training (HiT) data are given in green, yellow, and red, respectively. Data are given as mean ± standard deviation. Wellbeing status is given as TRUE if no negative marks like physical exhaustion, strain, or injury were reported. Otherwise, FALSE was given as well-being status.

POL (785 ± 71 au) revealed statistically significantly (*p* ≤ 0.001; SMD = 4.55) lower eTRIMP values (Figure 1A) than TRAD (1,273 ± 126 au) for the total 6-week training program. Training data (Figure 1B) revealed no between-group differences (*p* = 0.938; SMD = 0.03) in total training volume for POL (602 ± 20 min) and TRAD (601 ± 60 min). POL (472 ± 57 min) showed significantly (*p* ≤ 0.001; SMD = 7.39) higher LiT volume than TRAD (157 ± 29 min). In contrast, TRAD significantly (*p* ≤ 0.001; SMD ≥ 2.25) completed more ThT (POL: 78 ± 41 min vs. TRAD: 216 ± 71 min) and HiT (POL: 52 ± 19 min vs. TRAD: 228 ± 70 min) training volume. Thus, POL and TRAD revealed a training intensity distribution (LiT, ThT, and HiT) of 78.4%, 13.0%, and 8.6% and 26.1%, 35.9%, and 37.9%, respectively. A significant difference was observed in the comparison of wellbeing status between the POL and TRAD groups. The incidence of poor wellbeing, physical overexertion, or injury was significantly lower in the POL group than in the TRAD group, as indicated by Fisher’s exact test (*p*-value = 0.019). The odds ratio was 0.12, with a 95% confidence interval of 0.01–0.82, suggesting that participants in the POL group were less likely to experience adverse wellbeing outcomes.

### 3.2 Performance data

**FIGURE 2:**
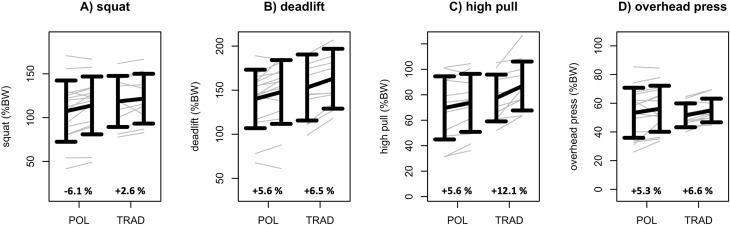
Squat 1RM **(A)**, deadlift 1RM **(B)**, high pull 1RM **(C)**, and overhead press 1RM **(D)** data of the polarized (POL) and the traditional (TRAD) HIFT training groups. Data are given as mean ± standard deviation. Individual data are given in gray. In addition, percentual mean change scores are given for POL and TRAD.

**FIGURE 3:**
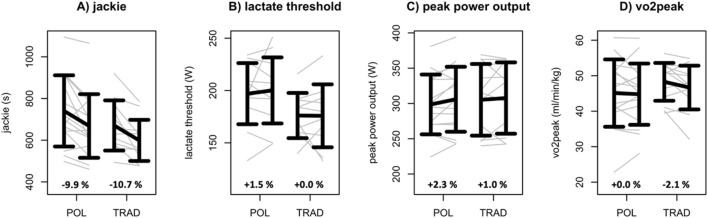
Jackie **(A)**, lactate threshold **(B)**, peak power output **(C)**, and V̇O_2_peak **(D)** data of the polarized (POL) and the traditional (TRAD) HIFT training groups. Data are given as mean ± standard deviation. Individual data are given in gray. In addition, percentual mean change scores are given for POL and TRAD.

Regarding the performance data, the 2 × 2 rANCOVA revealed no statistically relevant group × time interaction effects (*p* ≥ 0.094; η_p_^2^ ≤ 0.09) for all output parameters (Figures 2, 3; Table 2). In contrast, both groups revealed significant (rANCOVA time effects: *p* ≤ 0.02; 0.01 ≤ η_p_^2^ ≤ 0.11; 0.00 ≤ SMD ≤ 0.65) performance gains regarding DL1RM, OHP1RM, HP1RM, and Jackie.

**TABLE 2:** Performance data of the polarized (POL) and the traditional (TRAD) HIFT training groups.

Parameter	TRAD pre	TRAD post	SMD	POL pre	POL post	SMD	Time × group rANCOVA (*p* [η_p_^2^])	Time rANCOVA (*p* [η_p_^2^])
Jackie (s)	671 ± 120	599 ± 99	0.65	741 ± 171	668 ± 153	0.46	0.986 (0.00)	0.002 (0.01)
High pull (% body mass)	77.5 ± 18.4	86.9 ± 19.2	0.51	69.7 ± 24.8	73.6 ± 22.8	0.17	0.094 (0.09)	0.001 (0.11)
Squat (% body mass)	118.5 ± 29.1	121.6 ± 28.4	0.11	107.4 ± 35.0	113.9 ± 33.0	0.12	0.265 (0.03)	0.172 (0.02)
Overhead press (% body mass)	51.6 ± 8.3	55.0 ± 8.2	0.41	53.3 ± 17.4	56.1 ± 16.0	0.19	0.760 (0.00)	0.020 (0.00)
Deadlift (%body mass)	153.0 ± 37.5	163.0 ± 33.9	0.29	140.1 ± 33.2	148.0 ± 36.2	0.24	0.546 (0.02)	0.001 (0.02)
Lactate threshold (W)	176 ± 22	176 ± 30	0.00	197 ± 29	200 ± 32	0.10	0.681 (0.01)	0.961 (0.01)
Peak power output (W)	305 ± 51	308 ± 51	0.06	299 ± 42	306 ± 46	0.16	0.485 (0.02)	0.621 (0.02)
V̇O_2_peak (mL/min/kg)	48 ± 5	47 ± 6	0.20	45 ± 9	45 ± 9	0.00	0.328 (0.02)	0.100 (0.01)

Data are given as mean ± standard deviation. Effects of 2 (group: POL vs. TRAD) × 2 (time: PRE vs. POST) repeated measurement variance analysis with covariate (rANCOVA) interaction and time effects are given. rANCOVA effect sizes were given as partial eta-squared (η_p_^2^).

## 4 Discussion

This randomized controlled trial examined the effects of a time-matched polarized vs. traditional HIFT training regimen on relevant strength- and endurance-related performance outcomes. We found that relevant maximal strength, endurance, and CrossFit® related adaptations did not differ between groups, including performance gains in the deadlift, overhead press, and high pull strength and HIFT-specific exercises (Jackie benchmark workout).

Our main finding is that these similar adaptations were induced via a notably lower (about 40% less) total training load (eTRIMP) (Eather et al., 2016) in the polarized vs. traditional HIFT training group. In addition, our polarized training group reported relevant, less negative wellbeing notes than the traditional HIFT training group. This suggests that previously observed inadequate recovery, which can lead to undesirable effects such as decreased performance, disturbed sleep, increased perceived fatigue, and a higher incidence of respiratory tract infections (Hausswirth et al., 2019; Le Meur et al., 2013), via high amount of HIT might be reduced via the used polarized approach. However, it is important to note that proponents of HIT training do not recommend performing high volumes of HIT on consecutive days (Tibana et al., 2018), as it is suggested that a recovery period of 48–72 h is needed between full HIT sessions.

As strength and HIFT-specific performance gains via traditional HIFT indicated, HIT is a powerful stimulus in enhancing endurance performance (MacInnis and Gibala, 2017). Thereby, HIT requires a high energy turnover with an accumulation of reactive molecules and energy intermediates (Hawley et al., 2014). Subsequently, these metabolites accumulate and activate PGC-1alpha, which triggers mitochondrial biogenesis (Chandel, 2015).

At the same time, a significant percentage of HIT is a risk factor for adverse training effects, i.e., non-functional and functional overtraining (Meeusen et al., 2013). Particularly, traditional HIFT training revealed increased cortisol levels, which might indicate relevant stress in terms of fatigue and recovery demands (Faelli et al., 2020). Furthermore, a recent HIFT-related review (Jacob et al., 2020) revealed increased hormonal, metabolic, and inflammatory stress marker levels via traditional HIFT training. Therefore, our polarized HIFT training approach with a reduced amount of HIT might be useful to reduce such potential negative effects.

In the context of other endurance training (cross-country skiing, rowing, cycling, running, speed skating, and swimming), a recent review (Sperlich et al., 2023) revealed that successful athletes use either polarized or pyramidal training intensity distribution patterns characterized by a high amount (60%–90%) of LiT, with lesser amounts of ThT and HiT. However, regular incorporation of some high-intensity training is essential for optimal adaptation in motor units needed for competitive exercises (Foster et al., 2022). Thereby, the effectiveness of more polarized and pyramidal training, compared to threshold or high-intensity-based training, can be attributed to differential mitochondrial signaling pathways (Burnley et al., 2022; Foster et al., 2022) and potential adverse effects of excessive high-intensity training (Foster et al., 2022). Two primary signaling pathways for mitochondrial proliferation, one involving calcium signaling (associated with high-volume training) and the other involving AMPK signaling (linked to high-intensity training), converge on PGC1-α expression (Bishop et al., 2019; MacInnis et al., 2019; van der Zwaard et al., 2021). Thereby, a recent meta-analysis (Rosenblat et al., 2019) and review (Foster et al., 2022) supposed superior training effects of a polarized/pyramidal approach compared to threshold or high-intensity focused approaches. In contrast, our data revealed similar performance adaptations via polarized/pyramidal and threshold HIFT training. However, monotonous high-intensity training may disrupt homeostasis, causing inflammatory responses and delayed autonomic recovery (Meeusen et al., 2006; Seiler et al., 2007). These observations are supported by empirical evidence (Billat et al., 1999; Esteve-Lanao et al., 2007), indicating that excessive higher-intensity training may not be well tolerated. Our results regarding the well-being status might support these hypotheses.

We initially intended to compare polarized vs. traditional HIFT training. However, analysis of the training intensity distribution data showed that the polarized training group was better characterized by a pyramidal training distribution, and the high-intensity training group was better characterized by a threshold training distribution. This discrepancy could be partly explained by the heart rate-based time-in-zone approach employed in this study. Based on the delayed heart rate response to a HIT session (Plews et al., 2014), a time-in-zone method displays delayed heart-rate elevations and underreports time in HIT compared to a sessions-goal method (Sylta et al., 2014). Regardless, our traditional HIFT group revealed approximately 37% HIT, which is remarkably higher than the HIT amount of other endurance-related athletes (Sperlich et al., 2023). Despite, on average, the TRAD group being classified as THR and the POL group as PYR at the individual level, four participants from the TRAD group demonstrated a dominant HIT TID, while two participants from the POL group exhibited a polarized TID. Therefore, our data suggest that despite the same training program within groups, different TID patterns can emerge on an individual level.

Because the typical duration of polarized training interventions ranged from 4 to 16 weeks (Rosenblat et al., 2019), the short intervention period (6 weeks) of our study might be seen as a limitation. However, previous performance benefits of specific intensified training programs have been associated with interventions that are shorter than 8 weeks and mesocycles of comparable length (Billat, 2001; Ronnestad et al., 2016). To date, the benchmark workouts Karen, Fran, Grace, Helen, Filthy-50, and Fight-Gone-Bad have been examined in scientific publications (Mangine et al., 2018; Mangine et al., 2022; Tibana et al., 2022). None of those workouts were used, and only one HIFT-specific benchmark workout (Jackie) was used in this study. For better comparability, future research should consider integrating these previously examined benchmark workouts (Mangine et al., 2018; Mangine et al., 2022; Tibana et al., 2022). Moreover, the squat and V̇O2peak tests used in this study have previously been characterized as relevant performance surrogate parameters in HIFT (Bellar et al., 2015; Dexheimer et al., 2019; Martínez-Gómez et al., 2019; Meier et al., 2021; Zeitz et al., 2020). Another limitation concerns the method used to calculate the training load. Although we employed the heart rate-based method, Falk Neto et al. (2020) concluded that session RPE was more accurate than TRIMP-based methods to represent the overall training load of HIFT sessions. Apart from this, the repetition completion rate was also suggested as an easy and accurate tracking approach for intra- and inter-workout comparisons (Mangine and Seay, 2022). However, it should be noted that the concept of polarized training primarily uses heart rate-based approaches and not RPE or repetition completion rate (Seiler and Kjerland, 2006; Sperlich et al., 2023). Therefore, future research should investigate the integration and combination of heart rate-based methods with alternative approaches, such as those based on RPE or repetition completion rate. Finally, the current athletes revealed a relatively large variance regarding the corresponding output parameters, indicating a relevant heterogeneity of the sampling group. Therefore, future research should recruit more homogeneous groups of athletes, use longer intervention periods, and incorporate such RPE-based approaches.

In conclusion, the current data revealed similar performance adaptations via a time-matched polarized/pyramidal HIFT training approach compared to a threshold HIFT training approach. In addition, the polarized/pyramidal HIFT training approach was characterized by a substantially lower total training load (eTRIMP) (Edwards et al., 1994). Accordingly, a polarized/pyramidal HIFT training approach might be a promising option to reduce inadequate recovery. The practical applications of a polarized/pyramidal training approach in HIFT include optimizing training adaptation while minimizing recovery needs. This approach can be particularly beneficial for athletes seeking to maintain high performance levels over extended periods, as it allows for adequate recovery between high-intensity sessions. Future research could investigate these aspects and their effects when increasing the weekly training load using a workload-matched comparison between polarized/pyramidal and threshold HIFT training settings.

## Data Availability

The raw data supporting the conclusions of this article will be made available by the authors, without undue reservation.
