# Supplementary material for: Six weeks of polarized functional interval training with large training load reductions does not affect performance gains compared to traditional workouts
Source: Front Physiol. 2024 Nov 15;15:1446837. doi: 10.3389/fphys.2024.1446837 (PMC11604715; doi:10.3389/fphys.2024.1446837)
Supplement: Supplementary file 1 [file DataSheet1.pdf]

Supplementary Table 2: Overview of the training programm for the traditional high intensity functional interval training (TRAD).

**WEEK 1**	**Session 1**		**Session 2**		**Session 3**		**Session 4**	
**Strength**	3RM:<br>Back Squat<br>Hang Power Clean	1. Set: 10 reps, 1 min rest<br>2. Set: 6 reps, 1 min rest<br>3. Set: 4 reps 2 min rest<br>4. Set: 3 reps, 3 min rest<br>5. Set: 3 reps, 3 min rest<br>6. Set:	3RM:<br>Strict Press	1. Set: 10 reps, 1 min rest<br>2. Set: 6 reps, 1 min rest<br>3. Set: 4 reps 2 min rest<br>4. Set: 3 reps, 3 min rest<br>5. Set: 3 reps, 3 min rest<br>6. Set:	3RM:<br>Deadlift	1. Set: 10 reps, 1 min rest<br>2. Set: 6 reps, 1 min rest<br>3. Set: 4 reps 2 min rest<br>4. Set: 3 reps, 3 min rest<br>5. Set: 3 reps, 3 min rest<br>6. Set:	Pull Ups	5x ME, 2 min rest<br>sc: if < 3 reps do banded PU or inverted bar rows
**WOD / Endurance**	**3x 6min AMRAP, 4min rest**<br>**#1 AMRAP**<br>00:45 sec row<br>20 Air Squats<br><br>**#2 AMRAP**<br>10 alt. DB reverse Lunges @ 2x F:5-15kg / M:15 -22.5kg<br>10 Push ups / hand elevated Push ups<br><br>**#3 AMRAP**<br>20 DU / 60 SU<br>4 strict Chin Ups / Australian PullUps with supinated grip		**6x 3min on, 2min rest**<br><br>200m run (or row 1:00 if running is not possible for you)<br>10 single arm DB Thrusters (r 5/l 5)<br>in remainig time: max. reps Burpees<br><br>DB weight: @ F:5-15kg / M:15 -22.5kg		**4x 5min on, 3min rest**<br>**Set #1 und #3**<br>500m / 450m row<br>into AMRAP of:<br>10 Box Jumps @ F: 50cm / M: 60cm<br>10 Double DB Push Press<br><br>**Set #2 und #4**<br>500m / 450m row<br>into AMRAP of:<br>10 Double DB Box Step ups<br>10 alternating DB power Snatch<br><br>DB weight: @ F:5-15kg / M:15 -22.5kg		**start with: 400m run (or 800m cycle or 500/450m row)**<br>**6 min AMRAP 1:**<br>15/12 Cal Row<br>12 Box Jump<br>(3 Min Rest)<br><br>**6 min AMRAP 2:**<br>12/10cal Row<br>10 alt. DB Snatches @ F:5-15kg / M:15 -22.5 kg<br>(3 Min Rest)<br><br>**6 min AMRAP 3:**<br>10/8 Cal Row<br>8 DB Thruster @ F:5-15kg / M:15 -22.5kg<br>directly into<br>**finish with: 400m Run**	
**WEEK 2**	**Session 1**		**Session 2**		**Session 3**		**Session 4**	
**Strength**	Back Squat	3x8 @ 75% of 1RM (3RM x 0,825), 2min rest	Clean Pulls	4x5 @ 80% of 3RM Power Clean, 2 min rest	Strict Press	3x8 @ 70 of 1RM (3RM x 0,825)	Dips	5x RM, 2min rest; scaling:<br>if < 3 reps bench dips
**WOD / Endurance**	**6x3min on, 2min rest**<br><br>**#1, 3, und 5 AMRAP**<br>200m run (sprint)<br>5 DB Strict Press<br>10 Butterfly Sit ups<br><br>**#2, 4, und 6 AMRAP**<br>4 Burpees over DB<br>5 DB Front Squats<br>4 (banded) strict Pull ups/ Australian PullUps<br><br>DB weight: @ F:5-15kg / M:15 -22.5kg		**3x 6min AMRAP, 4min rest**<br><br>10 double DB Deadlifts<br>10 Toes to Bar / V-Ups<br>10 DB Squat Cleans<br>10 Burpee Box Jump over<br><br>DB weight: @ F:5-15kg / M:15 -22.5kg		**2x 5min AMRAP, 2 min off**<br>**AMRAP:**<br>Row M: 250/W: 200m<br>8 Push ups / elevated Push ups<br><br>**5x 2min AMRAP, 1min rest**<br>**AMRAP:**<br>15 double-unders / 30 Single Unders<br>8 Sumo Deadlift High Pull /w KB @8-12/16-20kg		**3x 8 min AMRAP 2 min rest**<br>**#1 AMRAP**<br>400m run<br>50 Air Squats<br>25 Burpees<br><br>**#2 AMRAP**<br>400m run<br>20 PushUps<br>10 Toes 2 Bar / Knees 2 Elbows<br><br>**#3 AMRAP**<br>400m run<br>20 Box Jumps<br>10 Burpee Box Jump Over	
**WEEK 3**	**Session 1**		**Session 2**		**Session 3**		**Session 4**	
**Strength**	1 hang power clean + 3 front squats	4x @ 80% of 3RM Power Clean	Strict Press	4x5 @ 75-80% of 1 RM (3RM x 0,9)	Deadlift	3x5 @ 80% of 1RM (3RM x 0,925)	Chin Ups	5x RM, 2min rest<br>if < 3 reps use a band<br>if > 8 reps use additional weight
**WOD / Endurance**	**4x 5min AMRAP, 2:30min rest**<br>**#1. und 2. AMRAP (5min)**<br>8 Burpees<br>12 DB Hang Power Cleans @ F:7,5-12 5 / M:15-22,5 (6l./6r.)<br>8 DB Bent Over Row @ F:5-15kg / M:15 -22.5kg<br><br>**#3. und 4. AMRAP(5min)**<br>250/200m row<br>12 Air Squats<br>8 Push Ups / hand elevated Push ups or 4 (kipping) Handstand Push ups		**6x 3min AMRAP, 2min Rest**<br>**#1. und 2. AMRAP (3min)**<br>10 DB Thruster @ F:5-15kg / M:15 -22.5 kg (5r/5l)<br>25 double-unders / 50 Single Unders<br><br>**#3. und 4. AMRAP (3min)**<br>10 DB Push Press @ F:5-15kg / M:15 -22.5kg (5r/5l)<br>10 T2B / K2E / V-Ups<br><br>**#5. und 6. AMRAP (3min)**<br>12 alt. DB Lunges @ F:5-15kg / M:15 -22.5kg (6r/6l)<br>15 Sit ups		**3x 6min AMRAP, 4min rest**<br>25 Jumping Jacks<br>10 KB Goblet Squat @ F:12-20kg/ M:16-24kg<br>6 Floor Press with 2x DBs F:7,5- 20kg / M: 10-25kg<br>15 Russian KB Swings @ F:12-20kg/ M: 16-24kg<br>4 strict Pull ups / Australian Pull Ups		**3x 6min AMRAP, 3min rest**<br>**#1 AMRAP**<br>200m run<br>20 alt. DB Snatches<br>10 Box Jumps<br>5 (kipping/butterfly)PullUps / Australian Pull Ups<br><br>**#2 AMRAP**<br>200m run<br>20 alt. DB Snatches<br>10 Box Jump Over<br>5 (kipping/butterfly)PullUps / Australian Pull Ups<br><br>**#3 AMRAP**<br>200m run<br>20 alt. DB Snatches<br>10 Burpee Box Jump Over<br>5 (kipping/butterfly) PullUps / Australian Pull Ups	
**WEEK 4**	**Session 1**		**Session 2**		**Session 3**		**Session 4**	
**Strength**	Back Squat	4x5 @ 85% of 1 RM (3RM x 0,925), 2:30 min rest	Clean Pulls	4x3 @ 80% of 3RM DL, 2min rest	Strict Press	4x4 @ 90-95% of 3RM, 2-3min rest	Ring Dips	5x 5-8 reps<br>if < 3 reps use a band<br>if > 8 reps use additional weight
**WOD / Endurance**	**6x 3min AMRAP, 2min rest**<br>**AMRAP 1, 3 and 5:**<br>start with 10 Burpees<br>then do an Amrap of:<br>8 Sit Ups<br>6 KB Swings<br><br>**AMRAP 2, 4 and 6:**<br>start with 10 Burpees to target<br>then do an AMRAP of:<br>8 alternating Box Step Ups with 2x DBs<br>6 Floor Press with 2x DBs<br>(use same weight for floor press and box step up)<br><br>KB: 12-24kg<br>DB: 7,5 - 22,5kg		**6 sets of 4min AMRAP, 2min rest**<br>**AMRAP 1, 2, and 3**<br>8x shuttle sprints (distance: 5-10m)<br>20 Deadlifts with 2x DBs<br>10 (hand elevated) Push Ups<br><br>**AMRAP 4, 5 and 6**<br>4x shuttle sprints<br>20 Goblet Squat with 1 DB<br>10m single arm DB Overhead Carry (each side)<br><br>DB weight: @ F:5-15kg / M:15 -22.5kg<br>use the same weight for each movement!		**3x 7min AMRAP, 3:30min rest**<br>ROW distance A, B or C<br>5 s.a. DB Squat Cleans (each side)<br>5 s.a DB Push Press (each side)<br>10 Box Jump over<br>5 s.a. DB Snatch (each side)<br>10 V-Ups<br><br>A for AMRAP 1: 500/450m row<br>B for AMRAP 2: 250/200m row<br>C for AMRAP 3: 150/100m row<br><br>DB weight: @ F:5-15kg / M:15 -22.5kg		**CrossFit Open Workout 23.1**<br>**14 Minute AMRAP:**<br>60cal row<br>50 Toes to Bar<br>40 Wall Balls (6/9kg)<br>30 Cleans (43/61kg)<br>20 Ring Muscle Ups<br><br>scaled Version:<br>60cal row<br>50 hanging knee raises<br>40 Wall Balls (4,5/6kg)<br>30 Cleans (30/43kg)<br>20 Pull Ups<br><br>(rest 5 Minutes)<br><br>8x 250/200m Row, 1min rest	
**WEEK 5**	**Session 1**		**Session 2**		**Session 3**		**Session 4**	

**Strength**	1 hang power clean + 2 front squats	4x @ 90-95% of 3RM Power Clean, 3min rest	Strict Press	4x3@ 90% of 3RM, 3min rest	Deadlift	3x3 @ 100% of 3 RM, 3min rest	strict Pull Ups	5x 5-8 reps + 10-15 ring rows, 2 min rest<br>if < 3 reps use a band<br>if > 8 reps use additional weight
**WOD / Endurance**	**AMRAP 9x 2min AMRAP - 1min Rest**<br>**#1 AMRAP**<br>6x 5-10m shuttle runs<br>10 V-up<br><br>**#2 AMRAP**<br>10 rev. Lunges w/ 2x DBs<br>5 Burpees over DB<br><br>**#3 AMRAP**<br>15 Air Squats<br>6 s.a. DB Strict Press each side<br><br>F: 7,5-12,5kg / M:15-22,5kg		**AMRAP 3 x 7min - 3min rest AMRAP**<br>25 double-unders / 50 Single Unders<br>10 russ. KB Swings<br>10 Push Ups / hand elevated Push ups<br>10 Sit ups<br>10 Goblet Squats w/ KB<br>remaining time: row for cals<br><br>F: 7,5-12,5kg / M:15-22,5kg		**8x Alternating 3min Amrap, 2min rest**<br>**AMRAP: 1,3,5 and 7**<br>10 Box Step ups with 2x DBs<br>6 (kipping/butterfly/strict) Pull Ups/ 10 Ring rows<br><br>**AMRAP: 2,4,6 and 8**<br>10 alt. DB Snatch<br>10 Burpees over DB<br><br>F: 7,5-12,5kg / M:15-22,5kg		**on a 20 minute running clock:**<br><br>**PART A)**<br>15min AMRAP5<br>burpee pull ups OR 5 burpees to target<br>10 shuttle runs (1 rep = 15m (7.5m out, 7.5m back))<br>*Add 5 burpee after each round.<br><br>**PART B)**<br>Immediately following Part A, athletes will have 5 minutes to establish:<br>1-rep-max thruster (from the floor)<br>use 2x DBs if you haven't done Thrusters with barbell before!	
**WEEK 6**	**Session 1**		**Session 2**		**Session 3**		**Session 4**	
**Strength**	Back Squats	4x3 @ 100% of 3RM, 3min rest	Clean Pulls	3x5 @ 3RM x 0,775, 2-3min rest	Strict Press	3x3 @ 100% of 3RM, 3min rest	Chin Ups	5x 5-8 reps, 3min rest<br>sc: if < 3 reps use a band<br>adv: if > 8 reps use additional weight
**WOD / Endurance**	**6x4min AMRAP, 2min rest**<br>**AMRAP 1, 2 and 3**<br>3 rounds of:<br>6 Push Ups / hand elevated Push Ups<br>8 alt. reverse Lunges with KB<br>THEN: in remaining time row for cals<br><br>**AMRAP 4, 5 and 6**<br>3 rounds of:<br>6 Burpees<br>8 Russian KB Swings<br>THEN: in remaining time row for cals<br><br>KB@ F: 8-20kg / M:16-28kg		**3x 7min AMRAP - 3min rest**<br>**#1 AMRAP**<br>175/150m Row<br>8 dbl. DB Floor Press<br><br>**#2 AMRAP**<br>20 alt. rev. Lunges<br>6 ChinUps / Australian PullUps<br><br>**#3 AMRAP**<br>20 double-unders / 40 Single Unders<br>10 V-Ups<br><br>@ F:5-15kg / M:15 -22.5kg		**8x alt. AMRAP: 3min work, 1:30 min rest**<br>**1# AMRAP 3min**<br>start with: 200m run<br>then Amrap of:<br>6 Toes to Bar / knee raises / Sit ups<br>6 double DB Deadlift<br><br>**#2 AMRAP 3min**<br>12 hang DB Snatch (6 each side)<br>6 alternating Box Step over with one DB<br><br>F: 7,5-15kg/ M:15-22,5		**20 Minute AMRAP:**<br>2 Rounds of:<br>5 wall walks / scaled wall walk<br>50 double-unders /Single Unders<br>Round 1: 15 snatches (weight 1) / alt. DB Snatch<br>Round 2: 12 snatches (weight 2) / alt. DB Snatch<br><br>2 Rounds of:<br>20 strict handstand push-ups / hand released push ups<br>50 double-unders / Single-Unders<br>Round 1: 9 snatches (weight 3) / alt. DB Snatch<br>Round 2: 6 snatches (weight 4) / alt. DB Snatch<br><br>Barbell weight:<br>♀ 29kg, 43kg, 56kg, 70kg<br>♂ 43kg, 61kg, 83kg, 102kg<br><br>Dumbbell weight:<br>♀ 7,5kg, 10kg, 12,5kg, 15kg<br>♂ 15kg, 17,5kg, 20kg, 22,5kg	

AMRAP (As Many Reps As Possible): A workout structure where participants perform as many repetitions of an exercise as possible within a set time frame; **EMOM** (Every Minute On the Minute): A training format where an exercise is performed at the start of every minute for a set number of repetitions. Any remaining time within the minute is used for rest; **DB**: Dumbbell; **RM** (Repetition Maximum): The maximum amount of weight a person can lift for a specified number of repetitions; **alt**. (Alternating): Refers to performing an exercise by alternating sides or limbs;
